# Effects of a gender-responsive maternal, newborn and child health program on health and economic outcomes during COVID-19 in Kenya: a mixed-methods study

**DOI:** 10.1186/s12939-025-02579-z

**Published:** 2025-09-30

**Authors:** Abiola Adeniyi, Justus E. Ikemeri, Alice Mũrage, Jeffrey N. Bone, Sheilah Chelagat, Gertrude Anusu, Anjellah Jumah, Sammy Masibo, Sam Mbugua, Michael Scanlon, Lauren Y. Maldonado, Violet Naanyu, Laura J. Ruhl, Astrid Christoffersen-Deb, Julia J. Songok

**Affiliations:** 1 Department of Obstetrics and Gynecology, UBC, Vancouver, Vancouver, BC Canada; 2 Population Health, Academic Model Providing Access to Healthcare, Moi University, Eldoret, Kenya; 3 Faculty of Health Sciences, Simon Fraser University, Vancouver, BC Canada; 4 Biostatistics, Clinical Research Support Unit, BC Children’s Hospital Research Institute, Vancouver, BC Canada; 5 Department of Health, County Government of Trans Nzoia, Kitale, Kenya; 6 Department of Medicine, Indiana University, Indianapolis, IN USA; 7 Department of Medicine, Massachusetts General Hospital, Harvard Medical School, Boston, MA USA; 8 School of Arts and Social Sciences, Moi University, Eldoret, Kenya; 9 AMPATH Consortium, Department of Pediatrics, Indiana University, Bloomington, IN USA; 10 Department of Obstetrics and Gynecology, University of Toronto, Toronto, Canada; 11 Department of Child Health and Paediatrics, Moi University School of Medicine, Eldoret, Kenya

**Keywords:** COVID-19 pandemic, Gender-responsive, Maternal health, Economic outcomes, Health equity, Social determinants of health, Kenya

## Abstract

**Background:**

The COVID-19 pandemic worsened health and economic disparities for women in resource-limited settings. Chamas for Change (*Chamas*, Swahili for ‘groups with a purpose’) is a gender-responsive maternal, newborn and child health program that combines health education with social support and microfinance activities to address systematic disparities in maternal and infant health outcomes. This study evaluated the program’s effectiveness in mitigating pandemic-related health and economic inequities in Trans-Nzoia County, Kenya, a region with significant pre-existing vulnerabilities.

**Methods:**

We conducted a mixed-methods study using an explanatory sequential design from March to December 2023. We collected quantitative data from 609 women in 3 cohorts: continuous *Chamas* participants (*n* = 128), discontinued (drop-out) participants (*n* = 240), and women without *Chamas* exposure (*n* = 241). We measured maternal health indicators and the Poverty Probability Index (PPI) score as primary outcomes. Quantitative analysis included linear mixed-effects models (unadjusted and adjusted). Qualitative data from focus group discussions and key informant interviews (*n* = 57) were analyzed using the Gender and COVID-19 Matrix.

**Results:**

Continuous *Chamas* participants achieved significantly higher rates of postpartum visits (OR = 19.54; 95% CI:3.76-101.57) and exclusive breastfeeding (OR = 8.04; 95% CI:1.52–42.43), demonstrating reduced disparities in essential maternal health services. They showed lower health insurance uptake (OR = 0.43; 95% CI:0.22–0.83) and minimal improvements in PPI scores. Qualitative findings revealed that while the pandemic disrupted health services, *Chamas* membership provided continuity of care through adapted CHW services. However, pandemic-related restrictions limited the program’s economic benefits, potentially due to the program’s shifted focus toward health service delivery during the crisis, intensifying existing economic inequities.

**Conclusion:**

The *Chamas* program effectively sustained maternal and child health practices during the COVID-19 pandemic through adapted CHW support but showed limited ability to protect members from economic hardship. This demonstrates both the resilience and limitations of community-based interventions during widespread crises. Our results highlight the need for robust governmental support and social protection measures to address underlying economic vulnerabilities for women. Future pandemic preparedness should integrate CHWs into formal health systems and focus on strengthening linkages with formal financial systems while supporting CHWs’ role in reducing inequities in maternal and newborn health service delivery during crises.

## Background

The COVID-19 pandemic posed unprecedented challenges to global health systems [1] with particularly severe consequences for vulnerable populations in resource-limited settings. In Africa, existing structural inequalities intensified these challenges [2, 3, 4, 5, 6]. In Kenya, the pandemic deepened disparities in healthcare access and gender equity, while creating widespread economic challenges [7, 8, 9]. Women experienced disproportionate negative effects because they served as primary carers, were over-represented in informal and service sector jobs, and took on increased unpaid domestic responsibilities [10].

Gender-responsive community-based programs offer potential buffer against health inequity [11, 12], leveraging theories of social capital, community resilience, and gender empowerment [13, 14, 15]. The Chamas for Change (*Chamas*) program in Western Kenya exemplifies these interventions. In 2012, the Academic Model Providing Access to Healthcare (AMPATH) in collaboration with Kenya’s Ministry of Health launched *Chamas* (Swahili word for groups with a purpose that traditionally referring to community savings groups). The program addresses health-related challenges for women, adolescent girls, infants and children through three core components: health promotion, group microfinance (Group Integrated Savings for Health and Empowerment or GISHE), and peer support [16]. The *Chamas* intervention leverages Community Health Workers (CHW), to provide health and social education to women during pregnancy and the first 1000 days of their child’s life. *Chamas* aims to improve women and children’s health, economic autonomy and empowerment through a 3-year curriculum. It aims to improve uptake of health services and behaviours, potentially reducing health inequities.

Studies have shown that empowerment and peer support significantly enhance Maternal, Neonatal and Child Health (MNCH) outcomes. Empowered women make informed health decisions, access essential services, and advocate for their rights and their children’s rights [17, 18, 19]. Peer support networks also mitigate the psychological effects of crises like pandemics, fostering resilience and well-being [20, 21]. Our previous research confirmed that *Chamas* participants showed improvements in MNCH indicators including increased facility-based deliveries (+ 8%), higher family planning uptake (+ 7.2%), and better child immunization rates (+ 15.6%) [16, 22, 23, 24, 25]. However, the evidence base remains limited regarding the effectiveness of such community-based interventions during large-scale health emergencies [3, 7, 8, 26, 27], particularly in low-resource settings [10, 28, 29, 30, 31].

We selected Trans-Nzoia County for this study due to its significant pre-existing health and socioeconomic disparities, which the COVID-19 pandemic likely intensified. With a population of 990,341, the county has a predominantly agrarian economy, with 82% of households engaged in crop farming [32, 33]. The county reports higher infant mortality (36 versus 32 deaths per 1,000 live births), adolescent pregnancy (18% versus 15%), and violence against women (41% versus 34%) compared to national averages [32, 33]. In addition, limited access to clean energy technologies (7% versus national 21%) reflects environmental vulnerabilities [32, 34]. These indicators highlight the county’s susceptibility to pandemic-related shocks and support its selection as a site for examining gender-responsive interventions.

Although scholars have emphasized the theoretical promise of women’s groups and peer-led models, few studies have empirically examined their effectiveness during systemic shocks such as the COVID-19 pandemic—especially in sub-Saharan Africa [35, 36]. This study examines how the *Chamas* program influenced women’s health and economic outcomes during the COVID-19 pandemic in Trans-Nzoia County, Kenya. Our mixed-method study addressed three key questions:

(i) Did *Chamas’* participants achieve better health and economic outcomes during the pandemic compared to non-participants?
(ii) What effect did continuing in the *Chamas* program have on health and economic outcomes during the pandemic?
(iii) Is the reported quantitative data on health-related and economic indicators consistent with the qualitative perceptions of the *Chamas* effect in Trans-Nzoia County?

We hypothesize that the program’s integrated approach - combining health knowledge, access to financial capital, peer support, and health service integration, buffered against pandemic-related disruptions. We compare outcomes among 3 groups: women who maintained *Chamas* participation, those who discontinued participation, and women without *Chamas* exposure. This study advances the discourse on gender-responsive crisis management in resource-limited settings, providing insights for future pandemic preparedness and response strategies aimed at reducing health inequities among vulnerable populations.

## Methods

### Study design

We used an explanatory sequential mixed methods design to capture both broad patterns and in-depth experiences of health inequities during the pandemic. This approach integrates quantitative and qualitative methods to provide a comprehensive understanding of complex phenomena [37]. We collected quantitative data to assess changes in health and economic outcomes among women, followed by qualitative data to understand how the *Chamas* program influenced these outcomes during the pandemic. We analyzed the data types separately and then used a convergent approach for the final integration and interpretation phase (Table 1).

**Table 1:** Study design for evaluating the Chamas

Study Phase	Step	Process	Outcomes
**Quantitative Phase**	a. Data Collection	609 women completed electronic questionnaire surveys, assessing the following: PPI Scores MNCH indicators Family planning uptake, Adequate antenatal visits, Facility Delivery Post-partum home visits Exclusive breast feeding Immunization uptake COVID-19 indicators COVID infection COVID Vaccination	PPI Scores MNCH indicators Family planning uptake, Adequate antenatal visits, Facility Delivery Post-partum home visits Exclusive breast feeding Immunization uptake COVID-19 indicators COVID infection COVID Vaccination
	b. Data Analysis	Descriptive and Inferential Statistics	Description of Chamas effects on economic and health outcomes
**Qualitative Phase**	a. Data Collection	57 Participants participated in 6 FGDs and 11 KII’s	Description of Chamas effects on economic and health outcomes
	b. Data Analysis	Thematic Analysis	Themes
**Mixed-Method Integration Phase**	a. Merging Quantitative and Qualitative Results	Combine quantitatively economic and health outcomes with qualitative themes	Joint display relating qualitative participant quotes to quantitative economic and health outcomes
	b. Interpretation	Use the integration of the data to yield a more comprehensive understanding of participants’ pandemic experience	Discussion

We used the Gender and COVID-19 Matrix, an analytical tool that captures gendered experiences during the pandemic and identifies varying impacts across socioeconomic groups [10]. This matrix contextualizes gender analysis through standardized frameworks, adopting a multidimensional and intersectional perspective on gender [38, 39]. It has been used in similar studies [40, 41], and provides a structured approach to gender analysis during crises. The framework examines six COVID-related domains: COVID risk, illness/treatment, health systems/services, social impact, economic impact, and security impact. For each domain, the matrix provides questions that explore how gender affects access to resources, decision-making power, and coping strategies. Our study focused specifically on the health systems/services domain (examining access, utilization, and quality of care) and the economic impact domain (examining income changes, financial coping mechanisms, and household resource allocation). This approach allowed us to systematically document how gender intersected with other factors such as age, education, and geographical location to shape women’s pandemic experiences.

### Chamas program description

Building on the program overview in the introduction, we provide additional operational details of the *Chamas* intervention. Bi-weekly group meetings facilitated by CHWs follow a structured curriculum covering essential MNCH topics including antenatal care, skilled delivery, postpartum care, exclusive breastfeeding, family planning, and child nutrition. For the GISHE component, members contribute flexible amounts to a group savings fund, which provides low-interest loans for health expenses, education costs, or small business investments.

Groups typically consist of 15–20 women who participate over a three-year period spanning pregnancy through the first 1000 days of their child’s life. The program differs from standard care through its integration of health education with economic activities, peer support structure, and community-based delivery rather than facility-based care. During the COVID-19 pandemic, the program adapted by implementing phone-based consultations, socially distanced home visits, and modified meeting protocols. Key implementation stakeholders include CHWs (facilitation and education), local health facilities (diagnostic and referral services), county health management teams (oversight), and AMPATH (technical assistance and monitoring).

We conducted our research in 4 sub-counties in Trans-Nzoia County, Kenya. The locations includes urban (Kitale in Saboti sub-county), peri-urban (Kiminini), and rural (Cherangany and Kwanza) areas all with poor health and socio-economic parameters [32, 42].

### Study participants

**Fig. 1:**
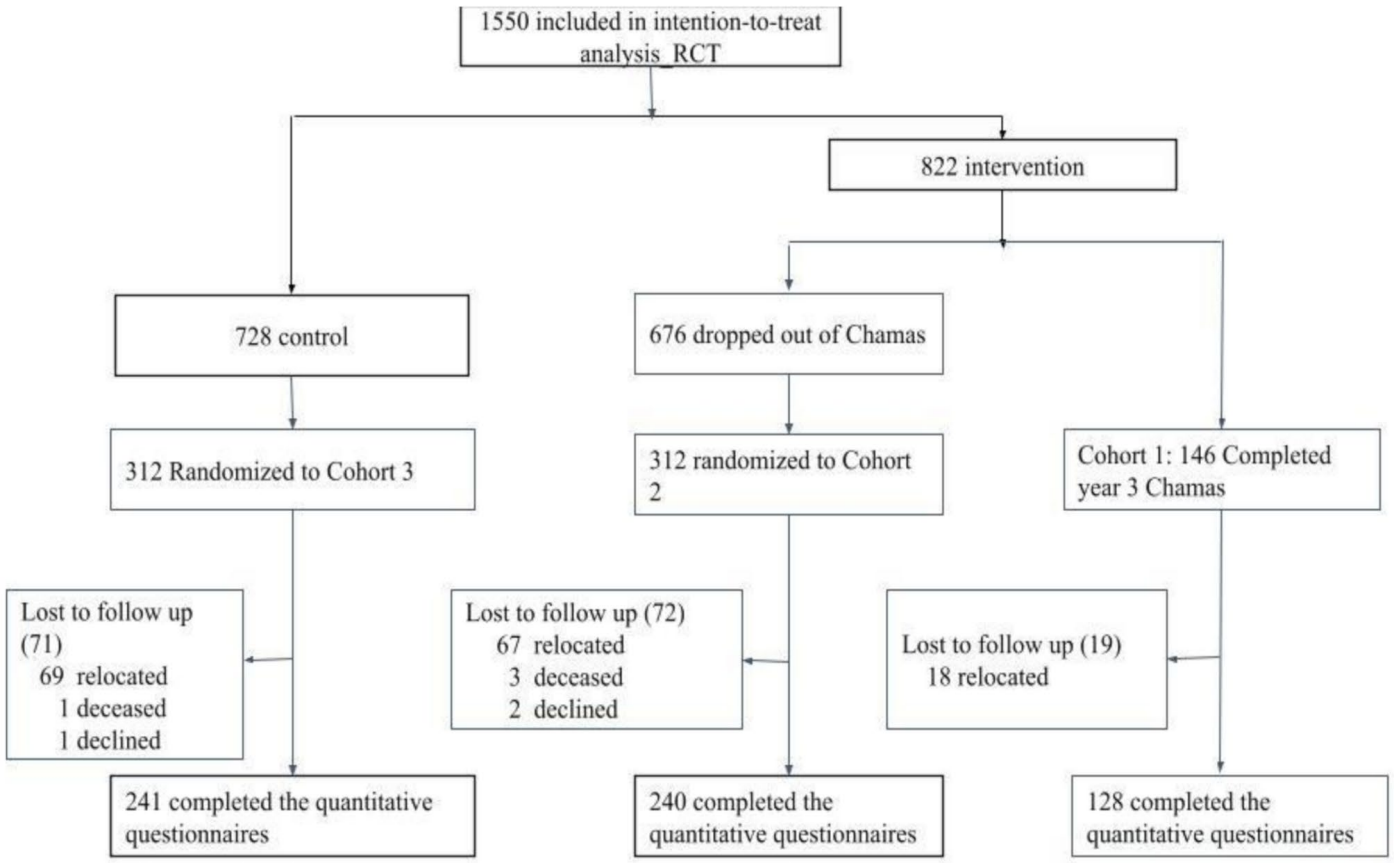
Chamas for change research participant flow diagram

We included women from our previous cluster randomized controlled trial (RCT) conducted from 2017 to 2019, involving 1,920 women from 74 community units in Trans-Nzoia County [23]. We identified 3 cohorts from the 1550 participants who completed the 12-month follow-up (Fig. 1): Cohort A includes 146 continuous *Chamas* participants active through December 2022, Cohort B consists of 676 participants who discontinued (defined as missing 3 consecutive *Chamas* meetings without communication or formally withdrawing from the program) before or during the pandemic (March 2020 - December 2022), and Cohort C comprising 728 women without *Chamas* exposure. We randomly selected 312 participants each from Cohorts B and C using a computer-generated random number sequence. After accounting for losses to follow-up (primarily due to relocation), our final sample included 609 women: 128 from Cohort A, 240 from Cohort B, and 241 from Cohort C.

We used purposive sampling across all three cohorts for the qualitative strand to intentionally select participants with diverse characteristics, knowledge, and experiences relevant to our research questions. We conducted focus group discussions (FGDs) with women from all cohorts (*N* = 21) pregnant and parenting adolescent girls (*N* = 16) who had participated in the pilot adolescent Chamas program, and women from the informal sector trade (*N* = 9) who were not categorized by *Chamas* participation. We also conducted key informant interviews (KIIs) with government officials, public health officers, and community leaders (*N* = 11) to understand institutional perspectives on program implementation and pandemic responses. This stratified sampling approach by age, education level, economic status, and geographical location allowed us to capture diverse experiences and perspectives across our study population. Interviews used English, Swahili, or local dialects based on participants’ preferences.

### Sample size

The Poverty Probability Index (PPI) served as our primary outcome measure. The median PPI score for women in our pre-pandemic RCT was 57 (SD = 21). We chose a 1:2:2 allocation ratio for follow-up among the 3 cohorts based on available resources and the limited number (146) who continued with *Chamas* during the pandemic. With a 0.5 correlation between pre- and post-pandemic PPI measures and the specified SD for a median PPI of 21, our study could detect a 6.5-point difference in post-pandemic PPI scores between groups, to achieve 80% power at a 5% Type 1 error level.

### Study variables

Our mixed-method study addressed three key questions previously outlined in the introduction: (i) comparison of health and economic outcomes between *Chamas* participants and non-participants during the pandemic; (ii) effects of continuous versus discontinued *Chamas* participation; and (iii) alignment of quantitative and qualitative evidence on the effects of the program. We developed an electronic questionnaire to gather demographic factors, health, economic, and psychosocial outcomes, focusing on pandemic health service utilization for pregnant individuals. We collected data on MNCH indicators, including antenatal care, facility delivery, 48-hour CHW visits, and exclusive breastfeeding, as well as COVID-19-related information such as infection rates and vaccine uptake, and enrolment in a government health insurance scheme referred to as the National Hospital Insurance Fund (NHIF). Adequate antenatal care attendance is defined as 4 or more visits, based on the Government of Kenya guidelines that were in effect before 2022 [43].

The qualitative strand explored participants’ experiences during the pandemic, highlighting *Chamas’* effect on women’s health and economic outcomes, along with the pandemic’s gender-specific effects. We developed interview guides based on the Gender and COVID-19 Matrix [10]. The research team drafted question guides for the FGDs and KIIs. The *Chamas* program’s Community Advisory Board (CAB) reviewed the tools for cultural appropriateness and relevance.

### Ethics

We obtained ethical approval from the International Development Research Centre (# 321/2022), the Kenyan National Commission for Science, Technology and Innovation (# P/23/30891), and the University of British Columbia (#H22-02812). The leadership of Trans-Nzoia County and community leaders approved the study. We got written informed consent from all participants after discussing the study’s risks, benefits, and time commitments. We used thumbprints and a literate witness for participants who could not read or write. We limited access to audio-recordings to relevant research team members to ensure confidentiality, de-identified subjects on transcripts, and stored recordings and subject keys in encrypted, password-protected computer files. All researchers who collected and analysed data completed ethical research training.

### Data collection procedures

From March to October 2023, we collected data through 2 streams. Trained CHWs and research enumerators administered electronic questionnaires using REDCap [44], hosted at AMPATH Kenya, for quantitative data. The *Chamas* implementation team supervised data collection conducted regular quality checks and provided ongoing support. Data collectors knew participants’ *Chamas* status, but did not know the study hypotheses to minimize bias. Interviews lasted 30–45 min in private settings. Participants received *Chamas* branded lesos (traditional African-print fabric) as culturally appropriate tokens of appreciation.

For qualitative data, we conducted 6 FGDs with 7–9 participants per group: 3 FGDs (one per study cohort including Cohort C the control group), 2 FGDs with pregnant and parenting adolescent girls (one with *Chamas* participants and another with non-participants) and one FGD with women engaged in the informal sector trade regardless of *Chamas* participation. Trained facilitators conducted 1–2 h sessions using semi-structured interview guides. We conducted key informant interviews (KIIs) with 5 health sector representatives, 5 local county government administrators and leaders and 1 representative from a collaborating non-governmental organization supporting maternal health initiatives in the region. We audio-recorded all sessions and produced verbatim transcripts. Two team members recorded field notes during each session to capture non-verbal cues and contextual details. All transcripts were translated to English for analysis. Participants were informed that anonymized results would be shared in publications and other dissemination activities.

### Outcomes

The primary outcome was the mean Kenya Poverty Probability Index (PPI) from 2015 score post-pandemic for the study participants. The PPI score is a validated tool that estimates the likelihood of a household living below the poverty line, using 10 questions about household characteristics and asset ownership [45]. Secondary outcomes included women’s healthcare utilization during the pandemic, COVID-19-related indicators (infection rates and vaccine uptake), enrollment in the NHIF, and MNCH indicators for pregnant individuals (specifically antenatal care, facility delivery, 48-hour CHW visits, and exclusive breastfeeding). We collected socio-demographic data such as age, marital status, education level, and occupation. The qualitative study explored participants’ experiences during the pandemic focusing on *Chamas’* effect on health and economic outcomes. We captured their perceptions on how *Chamas* shaped these outcomes and other nuances that quantitative data can miss.

### Data analysis

We summarized the characteristics of the 3 quantitative study cohorts with descriptive statistics, using medians and IQRs for continuous variables and counts and percentages for categorical variables. We descriptively compared participants in the follow-up study with those from the original RCT to assess potential selection bias. We used mixed effects models to assess outcomes differences at follow-up, incorporating random effects to account for data clustering. We used linear mixed effects models to analyse PPI scores, adjusting for RCT PPI score and logistic mixed effects models to compute odds ratios and 95% confidence intervals for binary outcomes. Models were fitted both unadjusted and adjusted for the following factors: age, education level, marital status, and baseline economic status. We assessed the subgroup of individuals who became pregnant during the follow-up period for delivery and pregnancy care-seeking behaviors. All analyses were conducted using R statistical software version 4.0.3.

We conducted a thematic analysis of the qualitative data, following the 6-step process by Braun and Clarke [46]: (1) Familiarization with the data through repeated reading (2) Systematic coding of interesting features across the dataset (3) Collating codes into potential themes (4) Reviewing themes for coherence and distinctiveness (5) Defining and naming themes (6) Producing the final analysis with compelling extract examples. Two researchers independently reviewed transcripts line-by-line, generating a code list and identifying main concepts from the data. This process included reviewing, reflecting, and re-reviewing the transcripts iteratively. The primary coding process clarified meanings, processes, and analytical categories. Ten coders conducted secondary analysis, refining codes and analyzing data with the Gender Analysis and COVID-19 Matrix to provide a gendered analysis and identify major themes. Data saturation was determined when no new themes emerged from additional interviews and focus groups; we began noticing repetition of themes by the 8th FGD but continued with remaining planned discussions to ensure adequate representation across all cohorts. We evaluated how additional data fit with preliminary themes and categories through constant comparison. We ensured rigor by employing member checking, presenting our preliminary findings to a subset of participants and the CAB for validation. We conducted analyses using NVivo 12 software.

We used an explanatory sequential design for data collection (quantitative followed by qualitative) combined with a convergent approach for the final integration and interpretation phase [47]. This allowed us to first establish patterns in health and economic outcomes, then explore the underlying reasons and experiences through qualitative inquiry and finally bring both datasets together for comprehensive interpretation. We compared quantitative data trends with themes from the qualitative analysis to interpret how the *Chamas* program impacted women’s health and economic outcomes during the COVID-19 pandemic. We used a joint display table to compare the quantitative and qualitative findings.

## Results

### Quantitative strand

Our study included 609 participants across 3 cohorts: continuous *Chamas* participants (Cohort A, *N* = 128), discontinued *Chamas* participants (Cohort B, *N* = 240), and non-participants (Cohort C, *N* = 241). Table 2 shows the demographic characteristics and key outcomes. COVID-19 infection rates were low (≤ 3%) with similar vaccination rates (≈ 50%) across cohorts. Healthcare utilization during the pandemic was reported by about 45% of participants in Cohorts A and B, and 40% in Cohort C, with no statistically significant differences. 58% of health service users reported greater difficulty accessing services. Table 3 shows minimal differences in the characteristics of participants in the control (Cohort C) and intervention (Cohorts A and B) groups from the original RCT study and the current investigation. Average PPI scores were similar across the 3 cohorts. Cohort A showed the lowest health insurance uptake (15%) compared to Cohort C (27%) (adjusted OR = 0.43, 95% CI: 0.22 to 0.83, *p* = 0.012). Conversely, Cohort A reported higher GISHE participation (97%) compared to Cohort B (67%) during active *Chamas* periods (adjusted OR = 16.74, 95% CI: 5.76 to 48.68, *p* < 0.001).

**Table 2:** Characteristics of the study population

Characteristics		Cohort A (*N* = 128)	Cohort B (*N* = 240)	Cohort C (*N* = 241)	Overall (*N* = 609)
**Participant Age [range]**		33.0 [28.0;36.0]	31.0 [26.0;36.0]	30.0 [25.0;36.0]	31 [25.0;36.0]
**Marital status**	Cohabiting (Not married)	0 (0.0%)	2 (0.8%)	1 (0.4%)	3 (0.5%)
	Divorced/separation	6 (4.7%)	18 (7.5%	12 (5.0%)	36 (5.9%)
	Married	114 (89.1%)	205 (85.4%)	212 (88.0%)	531 (87.2%)
	Single	5 (3.9%)	13 (5.4%)	14 (5.8%)	32 (5.3%)
	Widowed	3 (2.3%)	2 (0.8%)	2 (0.8%)	7 (1.2%)
**Education level**	College or higher	8 (6.3%)	12 (5.0%)	28 (11.6%)	48 (7.9%)
	Secondary or post-elementary	31 (24.2%)	58 (24.2%)	41 (17.0%	130 (21.4%)
	Elementary	48 (37.5%)	113 (47.1%)	109 (45.2%)	270 (44.3%)
	Pre-school or none	41 (32.0%)	57 (23.8%)	63 (26.1%)	161 (26.4%)
**Type of employment**	Informal sector	105 (82.0%)	178 (74.2%)	159 (66.0%)	457 (73.2%)
	Salaried job (Government/private job)	8 (6.3%)	7 (2.9%)	19 (7.9%)	34 (5.4%)
	Not working/unemployed	14 (10.9%)	51 (21.2%)	60 (24.9%)	125 (20.0%)
	Student	1 (0.8%)	4 (1.7%)	3 (1.3%)	8 (1.3%)
**Estimated households below poverty line**		23.1 [12.9;38.1]	22.6 [11.5;44.6]	21.6 [10.0;41.9]	
**Participating in non-GISHE group**	Yes	93 (72.7%)	134 (55.8%	126 (52.3%)	353 (57.9%)
	No	35 (27.3%)	106 (44.2%)	115 (47.7%)	256 (42.1%)
**COVID-19 infection**	Yes	4 (3.2%)	3 (1.3%)	4 (1.7%)	11 (1.8%)
	No	120 (94.5%)	234 (97.5%)	235 (97.5%)	589 (96.9%)
	Not sure	3 (2.4%)	3 (1.3%)	2 (0.8%)	8 (1.3%)
**Received COVID Vaccine (** ***n*** ** = 608)**	Yes	68 (53.5%)	118 (49.2%)	122 (50.6%)	308 (50.7%)
	No	59 (46.5%)	122 (50.8%)	119 (49.4%)	300 (49.3%)
**Sought health service during the pandemic (** ***n*** ** = 608)**	Yes	57 (44.9%)	109 (45.4%)	96 (39.8%)	262 (43.1%)
	No	70 (55.1%)	131 (54.6%)	145 (60.2%)	346 (56.9%)
**Ease of access to health services (** ***n*** ** = 346)**	Easy	27 (38.6%)	59 (45.0%)	60 (41.4%)	146 (42.2%)
	More challenging	43 (61.4%)	72 (55.0%)	85 (58.6%)	200 (57.8%)

**Table 3:** Summary descriptives of RCT study participants and current study participants

Descriptives		Control Current Study (*N* = 241)	Control RCT (*N* = 487)	Intervention Current Study (*N* = 368)	Intervention RCT (*N* = 454)
**Age**		26.0 [21.0;32.0]	25.5 [22.0;31.0]	28.0 [23.0;32.0]	25.0 [21.0;31.0]
**Parity**		2.00 [1.00;3.00]	2.00 [1.00;3.00]	2.00 [1.00;4.00]	2.00 [1.00;3.00]
**Gravida**		9.00 [3.00;11.0]	9.00 [3.00;10.2]	10.0 [9.00;11.0]	9.00 [3.00;11.0]
**Marital status**	Divorced/separated	5 (2.07%)	6 (1.23%)	6 (1.63%)	11 (2.42%)
	Married	204 (84.6%)	402 (82.5%)	320 (87.0%)	366 (80.6%)
	Single	32 (13.3%)	77 (15.8%)	40 (10.9%)	75 (16.5%)
	Widowed	0 (0.00%)	2 (0.41%)	2 (0.54%)	2 (0.44%)
**Highest Education**	College level or higher	33 (13.7%)	58 (11.9%)	21 (5.75%)	25 (5.52%)
	Pre-primary, none, or other	41 (17.0%)	72 (14.8%)	44 (12.1%)	58 (12.8%)
	Primary	102 (42.3%)	211 (43.3%)	206 (56.4%)	214 (47.2%)
	Secondary or post-primary, vocational	65 (27.0%)	146 (30.0%)	94 (25.8%)	156 (34.4%)
**Occupation**	Contract/temporary worker	14 (5.81%)	35 (7.19%)	20 (5.45%)	28 (6.17%)
	Permanently employed	7 (2.90%)	15 (3.08%)	2 (0.54%)	8 (1.76%)
	Self employed	70 (29.0%)	131 (26.9%)	119 (32.4%)	128 (28.2%)
	Unemployed	150 (62.2%)	306 (62.8%)	226 (61.6%)	290 (63.9%)
**Insurance**	I dont know	1 (0.43%)	5 (1.06%)	1 (0.28%)	2 (0.45%)
	No	91 (39.2%)	194 (41.2%)	133 (36.6%)	152 (34.2%)
	Yes	140 (60.3%)	272 (57.7%)	229 (63.1%)	290 (65.3%)
**Subcounty**	Cherangany	71 (29.5%)	158 (32.4%)	94 (25.5%)	117 (25.8%)
	Kiminini	54 (22.4%)	91 (18.7%)	74 (20.1%)	98 (21.6%)
	Kwanza	61 (25.3%)	132 (27.1%)	102 (27.7%)	114 (25.1%)
	Saboti	55 (22.8%)	106 (21.8%)	98 (26.6%)	125 (27.5%)
**PPI**		58.0 [39.0;70.8]	58.0 [45.0;69.0]	56.0 [38.0;68.0]	54.0 [39.0;69.0]

**Fig. 2:**
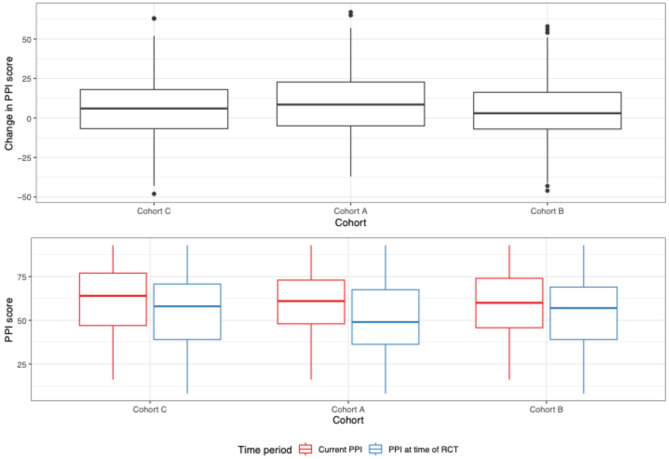
PPI Score and change in PPI score of RCT participants and current study participants

Table 4 presents key economic outcomes for the study cohorts. There were no observed differences in PPI between any of the 3 cohorts. Figure 2 shows the PPI and changes in PPI scores by cohort from the RCT period to the current study. Cohort A had slightly more variability in PPI score changes, while all cohorts had median changes near zero. Table 5 depicts MNCH outcomes for participants who were pregnant during the pandemic (*n* = 91). Cohort A had substantially higher odds of receiving a postpartum visit from a CHW within 48 h (OR: 19.54, 95% CI: 3.76 to 101.57, *p* < 0.001) and practicing exclusive breastfeeding for 6 months (OR: 8.04, 95% CI: 1.52 to 42.43, *p* = 0.014). Cohort A also had higher family planning rates, but lower recommended antenatal care (≥ 4 visits) completion (38%) than Cohorts B (57%) and C (63%). Facility-based delivery rates were similar across cohorts. Confidence intervals for comparisons of facility-based delivery, antenatal care, and family planning comparisons were very wide and no firm conclusions could be drawn.

**Table 4:** Economic outcomes for all participants

Economic Outcomes		Cohort A (*N* = 128)	Cohort B (*N* = 240)	Cohort C (*N* = 240)
**PPI Scores**	Median Poverty Probability Index score	56.5 [43.8;69.0]	57.0 [39.0;71.2]	58.0 [41.0;74.0]
	Mean Difference (95% CI)	0.34 (-6.32, 5.63)	-2.17 (-7.6, 3.26)	Ref
	Adjusted Mean Difference (95% CI)	0.05 (-4.34, 4.44)	-1.89 (-5.75, 1.96)	Ref
**Changes in PPI score** (pre vs. post pandemic)	Mean Difference (95% CI)	1.13 (-3.7, 5.96)	-1.59 (-5.86, 2.68)	Ref
	Adjusted Mean Difference (95% CI)	1.33 (-2.74, 4.44)	-1.39 (-4.93, 2.16)	Ref
**GISHE**	Participation (*n* = 608)	124 (96.9%)	161 (67.1%)	-
	OR (95% CI)	15.61 (5.49, 44.3)	Ref	-
	Adjusted OR (95% CI)	16.74 (5.76, 48.68)	Ref	-
**Insurance uptake**	Participation	19 (15.0%)	50 (20.8%)	64 (26.6%)
	OR (95% CI)	0.48 (0.26, 0.87)	0.73 (0.46, 1.17)	Ref
	Adjusted OR (95% CI)	0.43 (0.22, 0.83)	0.74 (0.44, 1.25	Ref
**Family planning uptake**	Participation	94 (73.4%)	185 (77.1%)	180 (74.4%)
	OR (95% CI)	0.82 (0.44, 1.54)	1.13 (0.65, 1.97)	Ref
	Adjusted OR (95% CI)	0.43 (0.22, 0.83)	0.74 (0.44, 1.25)	Ref

**Table 5:** MNCH outcomes in pregnant participants

MNCH outcomes		Cohort A (*N* = 13)	Cohort B (*N* = 44)	Cohort C (*N* = 34)
**Facility-based Delivery**	Participation	9 (69.2%)	32 (72.8%)	24 (70.6%)
	OR (95% CI)	0.94 (0.23, 3.76)	1.11 (0.41, 3)	Ref
**Adequate ANC Care (> 4 visits)**	Participation	5 (38.5%)	25 (56.8%)	22 (62.9%)
	OR (95% CI)	0.62 (0.12, 3.07	0.81 (0.24, 2.76)	Ref
**48 h CHW visit**	Participation	10 (76.9%)	16 (36.4%)	5 (14.7%)
	OR (95% CI)	19.54 (3.76, 101.57)*	3.33 (1.04, 10.67)*	Ref
**Exclusive Breastfeeding**	Participation	11 (84.5%)	26 (59.1%)	13 (36.1%)
	OR (95% CI)	8.04 (1.52, 42.43)*	1.95 (0.77, 4.96)*	Ref
**Family planning uptake**	Participation	12 (92.3%)	36 (81.8%)	23 (67.6%)
	OR (95% CI)	5.74 (0.66, 49.91)	2.15 (0.75, 6.15)	Ref

*- statistically significant

### Qualitative analysis

Table 6 summarises the characteristics of participants in the qualitative study. Our study cohort included 30 women from our 3 study cohorts and those involved in informal sector trade, 16 adolescents, and 11 key informants. Participants’ experiences and perspectives revealed 3 main themes that illustrate how the pandemic affected healthcare and economic outcomes in the county: (1) healthcare systems adaptation during COVID-19, (2) women bearing the economic burden, and (3) *Chamas* adaptation and COVID-19 resilience. These themes highlight opinions on the crisis’s effect and the resilience shown by women and communities during the COVID-19 pandemic.

**Table 6:** Demographic characteristics of qualitative study participants

Demographic Characteristics		Women (*N* = 30)	Adolescents (*N* = 16)	KII (*N* = 11)
**Mean Age (SD)**		36.4 (8.4	18.8 (1.0)	47.5 (9.19)
**Sex**	Female	30 (100.0)		2 (18.2)
	Male	0 (0.0)		8 (81.8)
**Marital status**	Cohabiting (Not married)	1 (3.3)	0 (0.0)	
	Divorced/separation	1 (3.3)	0 (0.0)	
	Married	27 (90.1)	1 (6.2)	
	Single	1 (3.3)	15 (93.8)	
	Widowed	0 (0.0)	0 (0.0)	
**Highest education**	College or higher	5 (16.7)	0 (0.0)	11 (100.0)
	Secondary or post-primary, Vocational	6 (20.0)	1 (6.2)	0 (0.0)
	Primary	8 (26.7)	15 (93.8)	0 (0.0)
	Pre-primary or none	11 (36.7)	0 (0.0)	0 (0.0)
**Occupation or Employment**	Informal sector	22 (73.3)	0 (0.0)	
	Formal sector (government/private job)	5 (16.7)	0 (0.0)	
	Student	0 (0.0)	12 (75.0)	

#### Theme 1: Healthcare system adaptations during COVID-19

This theme illustrates participants’ views on changes in healthcare access and delivery in Trans-Nzoia County during the COVID-19 pandemic, drawn from their experiences and observations. We identified 4 subthemes from the data: (1) health system constraints and financial barriers, (2) fear, stigma, and mental health issues, (3) disruption of maternal health services, and (4) emergence of alternative care strategies. These subthemes capture the systemic challenges that emerged during the crisis and the adaptive responses of healthcare providers and community members.

##### Health system constraints and financial barriers

Study participants described the pandemic’s strain on healthcare facilities and patient resources. Respondents opined that women faced increased costs and new payment requirements. One participant explained: *“In public hospitals they did not take cash*, *they wanted M-Pesa transactions. For family planning*, *the one for 100 shillings was now 300 shillings”* [FGD, Cohort C]. Several individuals shared stories of patients resorted to bribing healthcare workers: *“It* [Healthcare*] was also costly because you had to bribe*, *so that you can be attended to”* [FGD, Cohort C].

Healthcare officials described severe systemic constraints. A county health official outlined the challenges in resource allocation stating *“There was no budget for emergency… even Department of Health…”* [KII Health Official 1]. A key informant explained how local challenges were compounded by global supply chain disruptions: *“Even in the international market*, *some of these commodities were not there*, *because the demand was so high…I remember I was using people who used to import from China*, *and China had run out”* [KII County Administrator 1].

##### Fear, stigma and mental health issues

Many participants admitted avoiding healthcare facilities because they feared contracting COVID-19 and facing social stigma. One woman explained that: *“During corona even if you had malaria*, *we would not be willing to go to the hospital. We feared going there and be told we had corona hence go to quarantine”* [FGD, Cohort C]. Women described how individuals seeking care often faced stigmatization, as demonstrated by one participant’s experience: *“When they read my book they said*, *you have brought Corona for us. Even I was harassed a little before I was served”* [FGD, Cohort A]. Participants believed that the lack of support to frontline healthcare workers contributed to stigmatization: *“The people who were infected with COVID got victimized because the local health care workers had not been told how to deal with people COVID-19”* [FGD, Cohort B].

Healthcare providers shared experiences of fear and lack of adequate support systems. Officials highlighted what they perceived as the effect on service delivery: *“Doctor had that fear when they see maybe they can serve someone*, *and he has that disease. So*, *you get a doctor reaches in the morning and locks himself up in the hospital”* [KII Health Official 2]. Their accounts emphasized perceptions of healthcare access and heightened risks for women during the pandemic. From their viewpoint, women faced difficult choices between avoiding COVID-19 exposure and accessing essential care: *“…But you see for women [*who visited health facilities*] they’re putting themselves at risk”* [KII, Health Official 3]. Some health workers explained how healthcare delivery working conditions deteriorated significantly: *“…whereby you work*, *you respond to cases in the field*, *there is no lunch*, *there is no water*, *there’s no anything*, *it is just you individual*, *your own survival”* [KII, Health Official 2].

Focus group discussions indicated that parents and adolescents experienced particularly severe psychological impact. One participant described the combined effects of isolation and economic stress: *“Not going out made parents to become depressed. The parents were at home with no income and the children needed food”* [FGD, Cohort B]. Adolescents discussed their unique challenges, as highlighted in the FGDs: *“Me during COVID is when I got pregnant and that is when I got a lot of stress and I even wished to commit suicide because the parents have become tough”* [FGD, Adolescents, never been in *Chamas*].

##### Disruption of maternal health services

Women repeatedly mentioned healthcare services experienced major disruption. Their accounts described significant changes in maternal healthcare delivery during the crisis. According to these narratives, many experienced rushed deliveries and limited care. One participant described: *“When I went to give birth*, *I was released the same day. I gave birth at 5 p.m. and I went back home at 6 p.m.”* [FGD, Cohort A]. The discussions also highlighted that healthcare worker strikes during this time further limited access: *“The doctors had gone for a strike…You hear they have come back*, *you can come with your patient and when you arrive you hear*, *the doctors have gone for strike”* [ FGD, Adolescents, never been in *Chamas*]. Study participants described how medicine shortages created additional barriers: *“It wasn’t easy because you can go and be told that the medicine has run out*, *there are no injections*, *so you have to go back home”* [FGD, Cohort B].

##### Emergence of alternative care strategies

Participants detailed the development of alternative strategies to access care in response to these healthcare challenges. According to their accounts, many individuals sought assistance directly from local pharmacies: *“What I can say is that period people used to rely on going to the chemist they just go and explain to that person over there*, *and they write for him the medicines”* [FGD, Cohort C]. Their narratives suggested that traditional medicine gained new prominence: *“There is also the herbalists who used herbs. You go back to your home*, *and he bring it to you that medicine and tells you to take one cup of this medicine in the morning and in the evening”* [FGD, Adolescents, *Chamas*]. Stories emerged of some pregnant women postponing formal care in their pregnancy: *“… because I did not trust them. I did not go to the hospital. I went when I was 2 months due”* [FGD, Adolescents, never been in *Chamas*].

#### Theme 2: Women bearing the economic burden

This theme reflects participants’ perspectives on how women faced disproportionate economic challenges during the pandemic while showing their adaptability in response. The data revealed 3 subthemes: (1) job losses and reduced household income, (2) disruption of economic safety nets, and (3) women’s strategies for economic adaptation.

##### Job losses and reduced household income

Participants described significant economic disruption affecting women’s livelihoods. Job losses immediately strained household finances: *“… that time the parents were suspended from work and they came home and that is where they used to get money*, *so money became hard to find”* [FGD, Cohort C]. Many respondents reported how the economic pressure forced families to adopt survival strategies, including family separation, as one participant noted: *“Those who lived in towns had to send their families back to the rural areas due to the scarcity of money. This became the only way to survive”* [FGD, Woman, Cohort A]. Testimonies described how rising costs compounded these challenges: *“The cost of living increased and things prices increased and the money wasn’t there”* [FGD, Adolescents, *Chamas*]. A KII participant mentioned the pandemic’s lasting impact on county businesses stating: *“A lot of businesses were shut down. We even have some that could not come up even after the COVID pandemic have subsided”* [KII, County Official].

##### Disruption of economic safety nets

The data suggested that the pandemic severely affected the cooperative safety net women traditionally depended on. An NGO partner highlighted this stating: *“Women before COVID had a lot of things they were doing to elevate them economically*, *they had their chamas where they could collect money*, *loan each other… When COVID came*, *such stopped because now we [people] can’t meet”* [NGO Partner]. A county official highlighted this view: *“If we were to talk about a sector that was seriously affected*, *it was seriously de-organized by the COVID pandemic*, *this was the cooperative movement”* [KII, County Official].

##### Women’s strategies for economic adaptation

The FGD and interviews revealed that women developed innovative methods to generate income during the crisis. Many described assuming new roles to support their families: *“Or you can find like the father was doing work at a certain place and now he doesn’t have during that period*, *it is now up to the mother to look for a job like for example to look for kales [*a leafy green vegetable commonly grown and sold locally*] or spinach to come and sell”* [FGD, Cohort C]. Women recounted finding creative solutions through bartering, and small-scale enterprise: *“There are some of my friends who come to sew clothes*, *and some used to bring their fabrics*, *some have money*, *and others don’t have it so they give me maize”* [FGD, Cohort A]. Some proudly shared identifying new business opportunities in response to the pandemic, stating: *“I made masks and got rich with it”* [FGD, Cohort A].

#### Theme 3: Chamas adaptation and COVID-19 resilience

This theme highlights participants’ views on the evolution of support groups during the pandemic through 2 distinct subthemes: (1) the pandemic’s disruption of cooperative groups’ operations, and (2) the adaptation of *Chamas* group activities and support.

##### Pandemic’s disruption of cooperative groups’ operations

Study participants described how loan repayment challenges and reduced member contributions threatened the sustainability of cooperative groups. One member shared their experience: *“It affected us because we weren’t meeting and that time there were some who had taken loans. There are those who took loans and during Corona people ran away”* [FGD, Woman, Cohort A]. An NGO partner observed the effect on group resources: *“During that long period*, *even the resources they were using to do such activities*, *were used up maybe for food and all that*, *because that was now becoming more necessary”* [KII, NGO Partner].

##### Adaptation of *Chamas* group activities and support

Participants reported that *Chamas* groups showed adaptability by maintaining their operations during the pandemic. They detailed what they viewed as innovative solutions to continue their activities: *“We decided instead of stopping*, *let’s send money through the phone. We said if mganda [*table banking*] was in particular person’s house*, *we would send money then secretary and treasurer would confirm”* [FGD, Cohort A]. Another member described their adaptation strategies: *“We used to communicate and contribute our shares through the phone. It was hard to talk about Chamas and even ask for loans”* [FGD, Cohort B].

According to participants, *Chamas* groups served as vital sources of support for members during the crisis: *“COVID-19 came and became a challenge. So it provided a negative and positive impact to each and every individual. What I can say is that we learned more for we have known how to survive in hardship and we have known how to save in hardship”* [FGD, Cohort A]. Participants believed the groups helped combat stigma: *“The Chamas program provided better information and support*, *reducing the stigma associated with the disease”* [FGD, Cohort A]. One member proposed sustained support during crisis: *“My opinion to the government is*, *they can educate the people in the village on how to handle such an illness. They should put a certain amount aside there was a lot of hunger in the country”* [FGD, Cohort C].

### Integration of qualitative and qualitative findings

Our mixed-methods analysis revealed participants views of the influence of a community based maternal health program on health and economic outcomes during the pandemic as shown in Table 7. This integration allows us to better understand how the quantitative patterns were shaped by participants’ lived experiences during the crisis.

**Table 7:** Joint display of study findings

Key Finding	Quantitative Evidence	How Qualitative Data Explains the Pattern		Broader Implications
Higher postpartum care engagement but lower facility-based antenatal care in Cohort A	• Higher CHW postpartum visits in Cohort A vs. C • Lower facility-based antenatal care in Cohort A (39%) vs. C (63%)	• *Chamas* members relied on CHWs for care in pregnancy • CHWs provided phone and home visit support • Pre-existing relationships with CHWs enabled home-based care in pregnancy and postpartum	• Demonstrated how pre-existing trust relationships enable crisis adaptation • Shows how CHWs can maintain continuity of essential services during disruptions • Suggests need for better integration between community and facility-based care	
**Similar economic outcomes in all cohorts**	• Similar PPI scores across cohorts • Higher GISHE participation in Cohort A (97%) vs. B (67%) • Highest informal employment in Cohort A	• Widespread informal sector job losses • Disrupted community safety nets	• Reveals limitations of community microfinance during widespread economic disruption • Highlights need for stronger social protection systems • Suggests importance of linking community programs with formal financial institutions	
**Health insurance uptake lowest in Cohort A**	• Cohort C (27%) • Cohort B (21%) • Cohort A (15.0%)	• - Strong CHW relationships reduced perceived need for insurance • Regular CHW support provided alternative care access	• Indicates how economic pressures influence healthcare investment decisions • Suggests need for subsidized insurance programs during crises • Highlights tension between immediate survival needs and long-term health investments	
**Higher breastfeeding rates in continuous Chamas participants**	• Exclusive breastfeeding significantly higher in Cohort A (84.5%) vs. C (36.1%), OR = 8.04	• CHWs provided continued breastfeeding education and support • Economic pressures increased importance of breastfeeding as cost-saving measure	• Demonstrates resilience of behavior change interventions during crisis • Highlights economic factors in maternal health decision-making	

While Cohort A showed higher rates of postpartum CHW visits, they had lower facility-based antenatal care attendance. This pattern suggests the strong relationship between CHWs and *Chamas* members may have reduced the perceived need for facility-based care, particularly given COVID-19-related fears, and increased costs at facilities. Higher GISHE participation in Cohort A did not lead to any improved economic outcomes for this group, possibly due to widespread informal sector job losses and disruptions in financial support systems. The lower health insurance uptake among *Chamas* participants compared to non-participants might reflect reduced perceived need for insurance given their established relationships with CHWs for primary healthcare support. These findings suggest that while *Chamas* participation supported the maintenance of some maternal and infant health practices through CHW relationships, broader pandemic-related challenges affected the program’s overall impact during the crisis.

## Discussion

### Main findings and program effects

Our study examined how *Chamas* mitigated the pandemic’s effects on women’s health, and economic outcomes in Trans-Nzoia County, Kenya. The *Chamas* program demonstrated mixed effects. It effectively maintained maternal health practices through CHW engagement, especially in postpartum care, and exclusive breastfeeding. However, it struggled to protect members from economic hardship and related inequities. This pattern shows the program’s ability to overcome structural barriers through established trust and community relationships, while also highlighting its limitations in addressing deeper economic vulnerabilities during crisis.

Continuous *Chamas* participants showed significantly higher GISHE participation, but this did not lead to improved economic outcomes during the crisis. *Chamas* participants, despite higher rates of informal employment, achieved better postpartum care and breastfeeding outcomes through emergency-adapted CHW support strategies (phone consultations, socially distanced home visits, and modified group meetings). These findings align with studies showing CHWs effectiveness in addressing barriers to care during crises, particularly for vulnerable populations [48, 49, 50]. The program’s investment in community relationships and trust building enabled it to overcome traditional barriers to care during the crisis. CHWs maintained contact with members through these modified approaches, leveraging pre-existing relationships to create resilience in the health system. This relationship may have influenced *Chamas* members’ perceived need to attend ANC early or pay for NHIF. Economic factors and COVID-related fears limited participants’ engagement with facility-based antenatal care. These mixed results show how community-based interventions interact with broader societal inequities during crises [2, 8, 28].

### Strengths and limitations

Several strengths and limitations of our study warrant consideration. Our mixed methods approach provided comprehensive insights into how pre-existing socioeconomic differences affect program outcomes allowing us to capture both broad patterns and in-depth experiences. The inclusion of three well-defined cohorts enabled examination of both program participation effects and the impact of continued versus discontinued engagement. By applying the Gender and COVID-19 Matrix framework, we systematically captured intersecting factors affecting women’s experiences during the pandemic.

However, important limitations exist. Our cross-sectional design captures program adaptations during COVID-19, but limits assessments of long-term resilience effects. Without follow-up data, we cannot determine how program relationships may aided post-crisis recovery, or how the pandemic permanently altered program effectiveness. Self-reported data introduced potential bias, particularly during a period of considerable social disruption. The small sample of pregnant women during the pandemic (*n* = 91) requires caution in interpreting the maternal health outcomes. The qualitative findings do not generalize beyond our study population and social desirability bias may have influenced responses, particularly from participants who remained engaged in the program. The shift to remote program delivery and digital payments during the pandemic complicated our assessment of the program’s true effect, particularly due to varying access to digital resources among participants, which may itself represent an equity concern.

### Comparison with existing literature

When comparing our findings with existing literature, we found that our results align with studies on CHW’s effectiveness during crises [48, 49, 50]. The Chamas program’s success in maintaining postpartum care and exclusive breastfeeding during the pandemic parallels findings from CHW programs during Ebola outbreaks and natural disasters [51, 52]. However, our findings on economic outcomes contrast with some studies that have shown greater economic resilience from microfinance participation during crises [53, 54]. This difference may reflect the severity and widespread nature of the COVID-19 economic disruption compared to more localized crises examined in other studies. Our results more closely align with recent research showing the limitations of microfinance programs during the COVID-19 pandemic across multiple settings, particularly where informal employment predominates [53, 54].

Pregnancy is a vulnerable period for rural women with limited resources [55], a vulnerability which intensified during pandemic lockdowns. While *Chamas* attempted adaptation through phone-based services, the shift to digital payments and rising healthcare costs created additional barriers. The high rate of informal employment among continuous *Chamas* participants increased their vulnerability, highlighting how pre-existing inequities limit program effectiveness during emergencies. Notwithstanding, *Chamas’* participants high GISHE participation, similar PPI scores across cohorts demonstrate microfinance programs’ limitations during widespread economic disruption [56, 57]. Health insurance uptake also revealed a concerning pattern, as continuous *Chamas* participants exhibited significantly lower rates than non-participants. The lower health insurance uptake among continuous *Chamas* participants differs from our previous RCT results, which showed higher insurance enrollment among *Chamas* members [23]. This shift underscores how economic pressures during crises can reshape healthcare decision-making, forcing difficult choices between immediate needs and long-term health investments, a pattern also observed in other studies from low-resource settings during the pandemic. CHWs helped participants navigate these decisions, though structural barriers to formal financial services remained significant for those in informal employment [29, 31].

### Implications for policy and practice

Our findings have several important implications for practice and policy. First, they demonstrate that community-based health programs can maintain essential maternal and child health services during crises when they leverage existing trust relationships and adapt delivery methods. Health systems should integrate CHWs more formally into crisis response planning, providing appropriate support, training, and resources to enable continuity of care. Second, the limited economic protection offered by the GISHE component highlights the need for more robust social protection measures during widespread economic disruptions. Community-based microfinance programs should be complemented by formal social protection systems, particularly for workers in the informal sector who lack access to employer-based benefits or unemployment insurance.

Third, the lower health insurance uptake among continuous Chamas participants, despite their stronger engagement with CHWs, suggests that economic pressures during crises may force vulnerable populations to prioritize immediate survival needs over long-term health investments. This highlights the importance of subsidized or free essential health services during emergencies to prevent widening of health inequities.

The pandemic intensified gender inequities, as women assumed greater economic responsibilities and increased unpaid care work, irrespective of program participation [8, 28]. Findings from the qualitative data highlighted women’s resourcefulness in navigating structural barriers to develop income-generating strategies alongside household management. CHWs provided strong support for *Chamas* participants, despite movement restrictions, enabling higher postpartum visit rates and exclusive breastfeeding. This shows how community health programs can quickly adapt to crisis while maintaining essential services although challenges in facility-based care highlight the need for better integration into formal health systems [58, 59, 60]. Their role in maintaining maternal health practices during the pandemic shows how community-based programs can build resilient health systems, that protect against widening health disparities, especially with adequate institutional support. Future pandemic preparedness plans should incorporate CHW networks as a key component of resilient, equitable health systems.

### Future directions

Our findings indicate the need for a more comprehensive, gender-transformative approach to maternal and child health that addresses structural determinants of health inequity [61]. Programs need pre-established crisis response protocols and digital delivery mechanisms that account for varying resource access. During severe economic crises, suspending loan requirements and returning savings to participants may be necessary, as community-based lending faces limitations during widespread disruption. Programs should strengthen links to formal financial services and support participants in informal employment rather than relying solely on group-based mechanisms [61, 62].

Future research should employ longitudinal designs with larger sample sizes to better understand how economic factors, particularly informal employment status, influence the effectiveness of community-based interventions during crises and their effects on health equity. Studies should examine how programs can build preparedness through flexible delivery models and pre-established protocols, how financial commitments affect healthcare decisions during emergencies, and how gender norms influence program outcomes during crises. Policymakers must address the structural barriers that limit program effectiveness for the most vulnerable participants. Health systems must keep essential maternal health services accessible and affordable during emergencies, especially for women in informal employment.

Programs addressing maternal and child health during crises need a comprehensive, gender-transformative approach that considers pre-existing inequities. *Chamas* successfully maintained postpartum care and exclusive breastfeeding with the support of CHW indicating a model for crisis resilience that can help reduce care disparities. The similar PPI scores across cohorts despite high GISHE participation highlight the need for stronger government safety nets to protect vulnerable women during crises. Suggested improvements include graduated financial contributions based on economic capacity, strengthened linkages with formal financial services, and strategies to address digital barriers during crises.

## Conclusion

The *Chamas* program successfully maintained critical maternal health practices through adapted CHW support during the COVID-19 pandemic, demonstrating how community health worker relationships build resilience in maternal healthcare delivery systems and reduce disparities in access to essential services. Regardless of high GISHE participation among continuous participants, the program showed limited effectiveness in protecting members from economic hardship with comparable PPI scores across cohorts. This highlights the challenges in addressing underlying socioeconomic inequities during crises through community-based programs alone. Lower health insurance uptake among continuous pointed to the difficulties in maintaining long-term health investments during the crisis, particularly for women in informal employment.

Future pandemic preparedness requires programs that acknowledge women’s dual economic and caregiving responsibilities while providing flexible service delivery and challenging restrictive gender norms. Health systems must integrate these programs into formal structures and enhance crisis preparedness through comprehensive social protection. The *Chamas* experience provides a model for community health resilience and highlights areas for improving response. Future program development should leverage CHWs’ success in sustaining maternal health practices while creating stronger economic safeguards that address structural barriers during crises, particularly for women from disadvantaged socioeconomic backgrounds.

## Data Availability

The data that support the findings of this study are not openly available due to reasons of sensitivity and are available from the corresponding author upon reasonable request. Data are located in controlled access data storage at AMPATH Kenya.
